# Opioids in COVID-19: Two Sides of a Coin

**DOI:** 10.3389/fphar.2021.758637

**Published:** 2022-01-06

**Authors:** Camila Vantini Capasso Palamim, Matheus Negri Boschiero, Aléthea Guimarães Faria, Felipe Eduardo Valencise, Fernando Augusto Lima Marson

**Affiliations:** ^1^ Laboratory of Cell and Molecular Tumor Biology and Bioactive Compounds, São Francisco University, Bragança Paulista, Brazil; ^2^ Laboratory of Human and Medical Genetics, São Francisco University, Bragança Paulista, Brazil

**Keywords:** fentanyl, remifentanil, sufentanil, alfentanil, opioid use disorder and dependence, morphine, hydromorphone, methadone

## Abstract

**Introduction:** The treatment of most severe COVID-19 patients included the large-scale use of sedatives and analgesics–possibly in higher doses than usual–which was reported in the literature. The use of drugs that decrease mortality is necessary and opioids are important agents in procedures such as orotracheal intubation. However, these drugs seem to have been overestimated in the COVID-19 pandemic. We performed a review of the PubMed-Medline database to evaluate the use of opioids during this period. The following descriptors were used to enhance the search for papers: “Opioids”, “COVID-19,” “COVID-19 pandemic,” “SARS-CoV-2,” “Opioid use disorder,” “Opioid dependence” and the names of the drugs used. We also evaluated the distribution of COVID-19 patients in Brazil and the applicability of opioids in our country during the COVID-19 pandemic.

**Results:** Several positive points were found in the use of opioids in the COVID-19 pandemic, for instance, they can be used for analgesia in orotracheal intubation, for chronic pain management, and as coadjutant in the management of acute intensification of pain. However, high doses of opioids might exacerbate the respiratory depression found in COVID-19 patients, their chronic use can trigger opioid tolerance and the higher doses used during the pandemic might result in greater adverse effects. Unfortunately, the pandemic also affected individuals with opioid use disorder, not only those individuals are at higher risk of mortality, hospitalization and need for ventilatory support, but measures taken to decrease the SARS-CoV-2 spread such as social isolation, might negatively affect the treatment for opioid use disorder. In Brazil, only morphine, remifentanil and fentanyl are available in the basic health care system for the treatment of COVID-19 patients. Out of the 5,273,598 opioid units used in this period all over the country, morphine, fentanyl, and remifentanil, accounted for, respectively, 559,270 (10.6%), 4,624,328 (87.6%), and 90,000 (1.8%) units. Many Brazilian regions with high number of confirmed cases of COVID-19 had few units of opioids available, as the Southeast region, with a 0.23 units of opioids per confirmed COVID-19 case, and the South region, with 0.05 units. In the COVID-19 pandemic scenario, positive points related to opioids were mainly the occurrence of analgesia, to facilitate intubation and their use as coadjutants in the management of acute intensification of pain, whereas the negative points were indiscriminate use, the presence of human immunosuppressor response and increased adverse effects due to higher doses of the drug.

**Conclusion:** The importance of rational and individualized use of analgesic hypnotics and sedative anesthetics should be considered at all times, especially in situations of high demand such as the COVID-19 pandemic.

## 1 Introduction

The infection caused by the SARS-CoV-2 might affect different systems such as the gastrointestinal, central nervous, renal, cardiovascular and respiratory (Zhang et al., 2020). The most common symptoms include fever, cough, fatigue, and sputum production (Guan et al., 2020). At the same time, pneumonia associated with the COVID-19 might complicate due to the development of severe acute respiratory syndrome, and these patients might require admission in the intensive care unit (ICU), and be subjected to invasive mechanical ventilation (IMV) (Ammar et al., 2021).

In ICU patients under IMV, pain is one of the main reasons for restlessness, and moderate to deep levels of analgesia and sedation might be required as well as the use of neuromuscular blockade (NMB), to reduce the risk of cough, prevent asynchronous breath, and reduce the respiratory drive, which are harmful to the patient, and optimize ventilation, promoting suitable pain relief, and also preventing the activation of the sympathetic nervous system (Pandharipande et al., 2014; Allen et al., 2021; Ammar et al., 2021; Chaves-Cardona et al., 2021). Historically, the opioids are the most used class of drugs to perform sedation and analgesia in patients who need IMV. However, these drugs might be used carefully, since one of their most common side effects is the presence of respiratory depression, which can intensify the respiratory symptoms from COVID-19 such as shortness of breath (Roan et al., 2018; Ammar et al., 2021).

Even though the use of opioids might be necessary to help the ventilation of critically ill patients, prolonged use of sedatives in patients with respiratory insufficiency presents several adverse effects such as increase in hospital mortality, longer hospital treatment time, longer periods of IMV use and an dose dependent enhanced risk for delirium (Xing et al., 2015; Page, 2021). Additionally, the conditions described might indicate the patients’ worst prognosis and contribute to an increase in care costs, and interfere in their quality of life and survival rate after hospital discharge (Kotfis et al., 2020; Pun et al., 2021). It seems relevant to highlight that opioid have been widely used in critical COVID-19 patients under IMV. The literature suggests that patient subjected to IMV due to the COVID-19, often received higher doses of sedatives and analgesics when compared to patients with other clinical condition (Kapp et al., 2020; Page, 2021; Pun et al., 2021).

Another fact regarding this period is that the pandemic affected the individuals who already presented opioid use disorders in several different manners. For instance, recent studies observed that these individuals are at higher risk of SARS-CoV-2 infection, death, hospitalization, and need for ventilation (Baillargeon et al., 2021; Wang et al., 2021). Unfortunately, the impact of the COVID-19 was not limited to the worst outcomes of the disease. These individuals with opioid use disorder might be more susceptible to loss of income, isolation, lack of rewarding activities, fear and anxiety, which ultimately can enhance the risk of substance abuse (Columb et al., 2020; Khatri and Perrone, 2020; Mota, 2020; Henderson et al., 2021). One might also speculate that the pandemic provided less access to safe places to use opioids, leading to a high rate of overdose related calls to the paramedics (Galarneau et al., 2021). Thus, it is extremely important to revise the impact of opioid use during the COVID-19 in several aspects to improve the scientific evidence for other pandemics as well as to be prepared for the pos-pandemic period.

The objective of this narrative review was to discuss sedation and analgesia practices–particularly the use of opioids–in critical patients and the repercussion of these practices. It also aimed to carry out a review on the impact of the pandemic on individuals with opioid use disorder.

In this review, the PubMed-Medline database was surveyed regarding studies related to opioids and the COVID-19 published in the period from 2019 to 2021. The following descriptors were used to enhance the search for papers: “Opioids,”“Opioid use disorder,” “Opioid dependence,” “COVID-19,” “COVID-19 pandemic,” “SARS-CoV-2,” “SARS-CoV-2 infection,” and opioids [“Morphine”, “Oxycodone” “Fentanyl,” Hydrocodone,” “Methadone,” “Remifentanil,” “Sufentanil,” and “Alfentanil”]. Brazilian databases were also surveyed such as that made available by the Brazilian Health Ministry (https://covid.saude.gov.br/), to evaluate the Brazilian characteristics related to the COVID-19, including the number of confirmed cases, the number of deaths due to the COVID-19, incidence of the disease per 100,000 inhabitants, and mortality due to this disease per 100,000 inhabitants. Additionally, the study analyzed the distribution and number of opioids used all over the country according to the newsletter published by the Brazilian Health Ministry. We also estimated the total opioid use per confirmed COVID-19 cases, which was a ratio between total opioids and confirmed cases of COVID-19; and total opioids per death due to the COVID-19, which was a ratio between total opioids and deaths due to the COVID-19. In such scenario, we included a narrative review demonstrating the pros and cons of opioid use during the COVID-19 pandemic.

## 2 Results and Discussion

### 2.1 Physiological Effects of Opioids in COVID-19 and the Physiology of Dependence

Opioids might inhibit the release of neurotransmitters such as the Glutamate and the P substance released by the dorsal root ganglion at the level of the spinal and cerebral marrow through the activation of G proteins that inhibit the adenylate cyclase and regulate ionic canals through their bond to opioid receptors. In that context, three opioid receptors were established: mu, delta and kappa, which are metabotropic receptors that bond to the G protein, with different biomolecular structure, but with interrelated functions (Henriksen and Willoch, 2008; Bruijnzeel, 2009; Stein and Lang, 2009; Friedman and Nabong, 2020). These receptors can be found in high concentrations in supraspinal regions, such as the limbic area and regions related to neurohormonal secretion, as the hypothalamus, and most of these receptors are pre synaptic (Friedman and Nabong, 2020).

**FIGURE 1:**
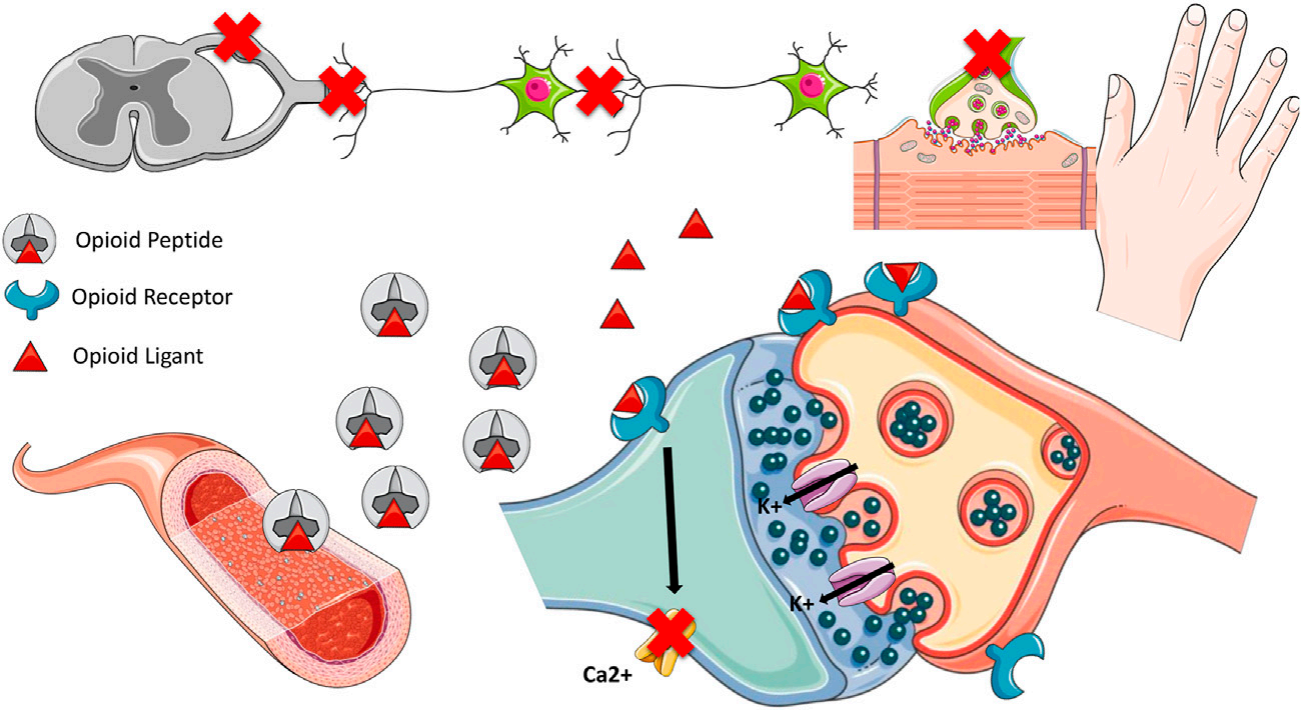
Pharmacodynamics of opioids. Opioids inhibit the release of Glutamate and Substance P by the dorsal ganglion neuron in the spinal cord and brain through the activation of G proteins, which inhibit adenylate cyclase and regulate ion channels by binding to opioid receptors. Once the opioid binds to the receptor, potassium influx and calcium channel blockage in the synaptic cleft occurs. Three opioid receptors: mu, delta and kappa, which are metabotropic receptors and bind to G protein, are responsible for the analgesic effect. Delta and mu receptor agonist opioids have mainly analgesic action, and delta receptor agonist opioids seem to present fewer side effects after a long period of use. The Kappa receptor can induce dopamine release and contribute to the development of hallucination and dysphoria behaviors. Opioids have a high volume of distribution due to their high liposolubility. Therefore, a short infusion bolus, for example, may have significant effects on plasma concentrations (Henriksen and Willoch, 2008; Bruijnzeel, 2009; Stein and Lang, 2009).

Agonist opioids of the delta and mu receptors present an analgesic action, while the agonist opioids of the delta receptor seem to present lesser side effects after long periods of use. Interestingly, the mu receptor is the main receptor for opioid agonists used in pain management (Friedman and Nabong, 2020). The kappa receptor, in turn, might induce dopamine release and cooperate with the development of hallucination and dysphoria behaviors, also, high concentrations of kappa receptors can be found in the spinal cord, and are thought to play a central role in the development of hyperalgesia. One can speculate that this might limit the development of drugs that interact with this receptor (Chavkin, 2011; Friedman and Nabong, 2020). Opioids show a high distribution volume and high liposolubility. Consequently, a short infusion bolus, for example, might have significant effects on plasma concentrations (Henriksen and Willoch, 2008; Bruijnzeel, 2009; Stein and Lang, 2009) (Figure 1). Moreover, some of these medicines present very short plasma half-lives such as the remifentanil and the alfentanil (Henriksen and Willoch, 2008; Bruijnzeel, 2009; Ammar et al., 2021).

**FIGURE 2:**
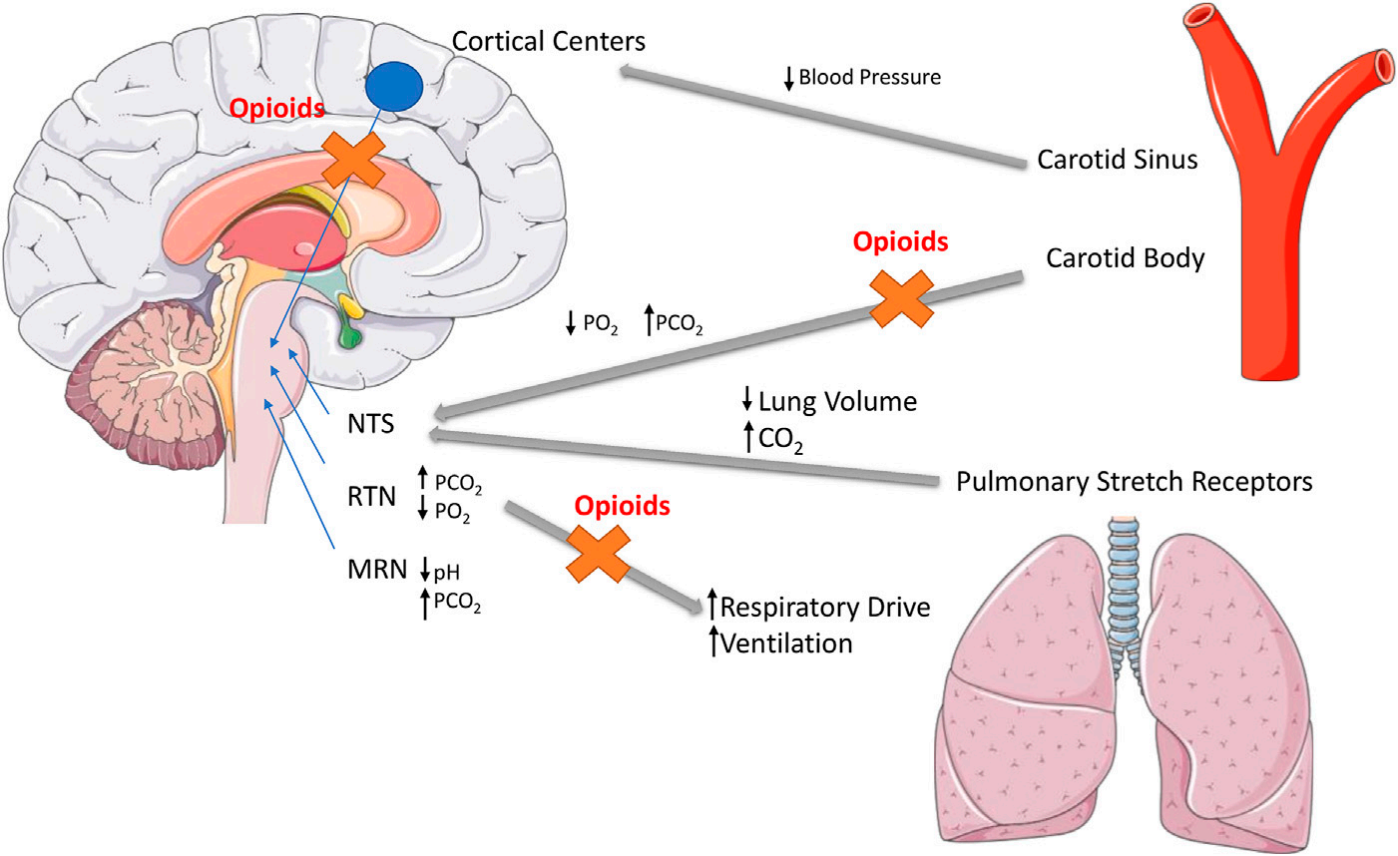
Opioid-induced respiratory depression mechanisms. Opioid-induced analgesia and respiratory depression arise from stimulation of μ-opioid receptors (MORs). MORs are expressed in neurons involved in the control of breathing, primarily located in the brainstem, particularly in the Nucleus Tractus Solitarius (NTS), Retrotrapezoid Nucleus (RTN) and Median Raphe Nuclei (MRN) (Boom et al., 2012).

Interestingly, the brainstream has a great concentration of Mu opioid receptors in areas involved with the control of breathing and the respiratory frequency, in which, if activated they may interfere of the process of breathing (Boom et al., 2012). Although the mechanism involved with respiratory depression is complex, opioids might increase hypercapnia and reduce tidal and minute volume, leading to slow and irregular breathing, which in severe cases can progress to fatal apnea (Leino et al., 1999; Boom et al., 2012). Furthermore, a great number of opioid receptors can be also found in the pre-Bötzinger complex, which is an important area related to the inspiration and has been recently described in humans. The activation of opioid receptors in this particular area might play a role in respiratory depression (Pattinson, 2008; Montandon et al., 2011; Schwarzacher et al., 2011; Boom et al., 2012) (Figure 2).

Unfortunately, opioids can also cause dependence due to their interaction with Mu receptors in the brain, resulting in activation of the reward mesolimbic system, which is also activated in several other daily activities such as sex and eating. The activation of the mesolimbic system, in turn, is responsible for the activation of the tegmental ventral area, located in the mesencephalon, which acts by releasing dopamine in the accumbens nucleus, which provides a feeling of pleasure (Kosten and George, 2002). Another factor that might result in dependence is the opioid action on the locus coeruleus. Normally, the locus coeruleus produces noradrenalin, an excitatory neurotransmitter that regulates several functions such as the respiratory frequency and blood pressure. However, opioids can act on the Mu receptors in this region, which reduces the noradrenalin secretion, leading to metabolic alteration that include reduced respiratory frequency and arterial pressure. As a consequence of the chronic ingestion of opioids, the locus coeruleus increases its noradrenalin secretion in an attempt to manage the opioid effect. Therefore, when a reduction in the concentration of opioids in the nervous system occurs and greater noradrenalin concentration is observed, several symptoms of the withdrawal syndrome such as anxiety and the presence of muscle cramps might appear (Kosten and George, 2002).

Regarding the physiological effects of opioids, we observed several positive points, as the mechanisms involved in analgesia, and those involved in the IMV. However, some negative points were also observed such as chest wall rigidity, which can increase the respiratory depression, and the mechanism related to opioid dependence.

Additionally, even if opioids belong to the same class of drugs, they present distinct pharmacodynamic, pharmacokinetic mechanisms and molecular structure (Table 1)**.**

**TABLE 1:** Characteristics of the main opioids used in patients affected by the coronavirus disease (COVID)-19. Adapted from Ammar et al., 2020.

Medication	Mechanism of action	Pharmacokinetics	IC50	EC50	Potency^a^	Adverse events	Place in therapy	Patients care considerations	Available at SUS
Fentanyl	Mu-opioid receptor agonist	(i) Onset: immediate	<20 nM	1.58 ± 0.04 nM	80–100×	Chest wall rigidity with rapid infusion	First-line therapy	(i) Prolonged and unpredictable clearance can be extended beyond infusion discontinuation	Yes
		(ii) Duration 3–60 min						(ii) Risk of hypotension lower than morphine	
		(iii) T1/2 > 100 min						(iii) Accumulation in hepatic dysfunction	
		(iv) Elimination T1/2: 2–4 h						(iv) Fentanyl patch is an alternative, but consider absorption (delayed onset and offset) and effect issues	
Morphine	Mu-opioid receptor agonist	(i) Onset: 5–10 min	193 nM	50–100 nM	1x	Hypotension and bradycardia	First-line therapy	(i) Metabolite can accumulate in kidney dysfunction	Yes
		(ii) Duration: 3–5 h						(ii) Accumulation of morphine-6-glucorinide and morphine-3-glucorinide can cause neurotoxicity	
		(iii) Elimination T1/2: 3–4 h						(iii) Enteral morphine is an alternative during shortage	
Hydromorphone	Mu-opioid receptor agonist	(i) Onset: 15–30 min	>50 μM	>0.41 nM	0.9–1.2 mg is equivalent to 10 mg morphine	Hypotension	First-line therapy	(i) 5–7 times more potent than morphine	No
		(ii) Duration: 3–4 h						(ii) Accumulation of hydromorphone-3-glucoronide in kidney dysfunction can cause neurotoxicity	
		(iii) Metabolized into hydromorphone-3-glucorinide							
		(iv) Elimination T1/2: 2–3 h							
Remifentanil	Mu-opioid receptor agonist	(i) Onset: 1–3 min	0.19 nM	30 nM	100–200x	Hypotension and chest wall rigidity	Alternative therapy	(i) Monitor for opiate withdrawal symptoms for 24 h after discontinuation	Yes
		(ii) Duration: 3–10 min						(ii) No accumulation in hepatic/renal failure	
		(iii) Offset: 5–10 min						(iii) Can cause serotonin syndrome with concomitant use with serotonergic agents	
		(iv) Terminal T1/2: 10–20 min (v) Metabolized by blood and esterase							
Sufentanil	Mu-opioid receptor agonist	(i) Onset: 1–3 (IV) and 30 min (sublingual)	5.5 nM	1.8 ± 0.8 nM	500–1000×	Bradyarrhythmia and hypotension	Alternative therapy	(i) Can cause serotonin syndrome with concomitant use with serotonergic agents	No
		(ii) Duration: 2 h (IV) and 3 h (sublingual)						(ii) 5–10 times more potent than fentanyl	
		(iii) T1/2: >100 min (IV) and 3 h (sublingual)							
Alfentanil	Mu-opioid receptor agonist	(i) Onset: 5 min	2.5 nM	1,248 ± 391 nM	8–20×	Hypotension	Alternative therapy	(i) 5 times more potent than fentanyl	No
		(ii) Duration: 30–60 min (iii) T1/2: 1.5–2 h						(ii) Can cause serotonin syndrome with concomitant use with serotonergic agents	
Methadone	Mu-opioid receptor agonist and NMDA receptor agonist	(i) Onset: 0.5–1 h (PO) and 10–20 min (IV)	NI	NI	150×	QTc prolongation	Opioid conservation and adjuvant therapy	(i) Long half-life	No
		(ii) Duration: 12–48 h						(ii) Prolonged effect with hepatic and renal dysfunction	
		(iii) T1/2: 8–59 h						(iii) Elimination half-life does not match short duration of analgesic effect	
		(iv) Reaching steady state in 3–5 days						(iv) Caution with administration of other drug which can enhance QTc prolongation	

IV, intravenous; PO, per oral; NMDA, N-methyl-D-aspartate receptor; QTc, corrected QT, interval; IC50, half the maximum inhibitory concentration; EC50, concentration of a drug that gives half-maximal response; NI, not informed; SUS, Sistema Único de Saúde - Brazilian Public Health System; T 1/2, half-life; μM, micromolar; nM, nanomolar; mg, milligrams.

a Potency is compared to morphine.

Adapted from (Ammar et al., 2021).

References: [Mahler and Forrest, 1975; Villiger et al., 1983; Yu and Sadée, 1988; Martin et al., 1991; Chiu et al., 1993; Lambert et al., 1993; Gozzani, 1994, 1994; Fantoni et al., 1999; Lötsch, 2005; Vieweg et al., 2005; Hannam et al., 2016, 2; Jeleazcov et al., 2016; Li et al., 2017; Palladone capsules 1.3 mg—Summary of Product Characteristics (SmPC)—(emc)]

### 2.2 Opioids Used in Patients’ Sedation

Pulmonary impairment is one of the main pathophysiological mechanisms of the COVID-19. Patients with this disease might present pain and suffering, not only due to the illness, but also as a result of invasive procedures such as the IMV, required by around 69% of the COVID-19 patients admitted in ICU (Devlin et al., 2018; Ammar et al., 2021; Chang et al., 2021). Analgesia, mainly using opioids, in this type of patients becomes usual, in order to provide them with comfort and also enable the accomplishment of further procedures such as orotracheal intubation (Allen et al., 2021). In the literature, opioids such as fentanyl, morphine, and hydromorphone are the main drugs used to treat ICU patients (Ammar et al., 2021). Our review summarizes the characteristics of the main opioids used in the treatment of COVID-19 patients (Table 1).

Fentanyl outstands as the most used opioid in the analgesia of conventional diseases. However, it is necessary to be cautious when using it through intravenous administration, since one of its main adverse effects is chest wall rigidity increase leading to respiratory depression (Roan et al., 2018; Ammar et al., 2021), which is recurrent in COVID-19 patients. Another drug that can be used to alleviate the discomfort caused by dyspnea is morphine (Ammar et al., 2021). Hydromorphone, in turn, can be used to substitute morphine or fentanyl, whenever the health service does not have the other medications, however, this opioid presents higher dosage error rate, when compared to other opioids, for this reason, health professionals must use it with caution to prevent overdoses of this medication (Ammar et al., 2021).

Other options of opioid analgesics for the treatment of COVID-19 patients include remifentanil, sufentanil, and alfentanil, which are drugs used in the hospital practice. However, they show some limitations that reduce their use in large scale situations. Remifentanil is associated to higher risk of hypotension, when compared to fentanyl, and has a shorter half-life, which might reduce the duration of its analgesic effect. Sufentanil and alfentanil are less frequently used in ICU also due to their short half-life. In addition, sufentanil might accumulate progressively when used in continuous and prolonged infusions. As for alfentanil, there are few reports of its use in continuous infusion by intensive care teams (Egan et al., 1993; Joshi et al., 2002; Ammar et al., 2021). However, these drugs are still considered options when the most commonly used drugs (morphine, hydromorphone, and fentanyl) are not available in the health service.

The advantages observed include the fact that many opioids such as fentanyl, hydromorphone, morphine, sufentanil, remifentanil, and alfentanil can be used in order to help in the IMV, and they are important to manage COVID-19 patients. However, since fentanyl is the most used opioid, the health care personnel might not have experience with the others, which might lead to dosage error. Also, sufentanil, remifentanil, and alfentanil show more limitations when compared to fentanyl, since they have a shorter half-life.

### 2.3 Opioids in Brazil: Availability, and Dependence

When managing COVID-19 patients, few drugs presented proved efficacy to modulate the outcome mainly regarding more severely affected individuals that required intensive care treatment and IMV. Among these drugs, dexamethasone and remdesivir reduced mortality risk and hospital care time, respectively (Beigel et al., 2020; RECOVERY Collaborative Group et al., 2021). However, other drugs such as opioids gained relevance in the COVID-19 pandemic for providing patients with greater comfort during treatment. Another fact to be taken into consideration is that since the start of the pandemic, Brazil has supported the acquisition of several drugs without scientific evidence for the COVID-19 treatment such as hydroxychloroquine, chloroquine and oseltamivir (Boschiero et al., 2021; MS-SUS COVID-19 Medications) spending around BRL 90 million to purchase such drugs (MS-SUS COVID-19 Medications). Curiously, the amount spent could have been used in the acquisition of other medicines, including opioids, which were missing in many healthcare centers in several parts of the country at certain times during the pandemic. As a result of the magnitude of the COVID-19 pandemic in Brazil, with approximately 22 million confirmed cases and over 600 thousand deaths [WHO Coronavirus (COVID-19) Dashboard] a variety of medicines, mainly opioids, were used to manage patients in ICU and under IMV.

In Brazil, around 80% of the population is assisted by the National Unified Health System (SUS, the Brazilian public health system), while the remaining population use private health care. Curiously, SUS is responsible for only 45% of the total expenditure with health in the country, while the private system accounts for 55%, this fact disagrees with the volume of assistance provided in each health sector (public and private) (SUS—20 years, 2021). Unfortunately, according to the Relação Nacional de Medicamentos Essenciais - Rename (Essential Medication National List), when it comes to opioids, only morphine and fentanyl are available for routine use at the SUS, and the small variety of drugs available can be explained, at least partly, by the low investment in this service (Rename, 2020). Therefore, the fact that the SUS that assists most of the population does not have enough resources to assist suitably those that requires this service is a matter of concern, mainly in a public health emergency situation such as that provoked by the COVID-19 pandemic.

As a consequence of the high use of opioids during the COVID-19 pandemic and public resource bad management, mainly by the federal government, there were reports of lack of opioids, as well as shortage of other medicines and inputs needed to perform intubation in Brazilian patients (Boschiero et al., 2021; Folha de São Paulo, 2021); and there were several reports of collapse in the health service. For example, according to the Associação Nacional de Hospitais Privados–ANAHP (Private Hospital National Association), on March 18, 2021, the institutions that are members of that association reported having a stock of fentanyl that would last only 20 days (ANAHP, 2021). Also, according to a survey carried out up to April 13, 2021 by the Federação das Santas Casas e Hospitais Beneficentes do Estado de São Paulo–Fehosp (Federation of Santa Casas and other charitable hospitals of São Paulo), around 160 hospitals had stocks of anesthetics and other medication needed for intubation that would only last from 3 to 5 days, with certain municipalities such as Guarujá and Rio Preto reporting even lower stocks that would probably end in 2 or 3 days (Fehosp–News). Such supply crisis affected and might still affect the combat to the pandemic in Brazil, preventing the treatment of patients that require intubation and potentially increasing dosage errors by the medical team, for not being acquainted with the use of the alternative medication available (Adams et al., 2020) or even, impairing the analgesia of those patients, preventing measures to alleviate their respiratory distress.

Unfortunately, the medication supply crisis in Brazil goes beyond opioids, several means of communication informed and are still informing that hospitals have low stocks of the “intubation kit,” that is, medication and necessary supplements to carry out orotracheal intubation (CNM, 2021; Folha de São Paulo, 2021). This fact might have contributed, at least partly, to the high mortality rate of patients in ICU throughout the country. In fact, the mortality rate among Brazilian patients with the COVID-19 disease in ICUs (∼55%), was higher than those of many other countries such as China (37.7%), Italy (25.6%), Spain (29.2%), United States of America (23.6%), Denmark (41.2%), Germany (24.3%), and the United Kingdom (8.0%) (Quah et al., 2020; Ranzani et al., 2021). The figures in Brazil were distributed differently among the states and regions of the country, with the highest death index, 79%, being observed in the Northern region of the country (Table 2).

**TABLE 2:** Epidemiological characteristics of coronavirus disease (COVID)-19 cases, death, and distribution of opioids in the Brazilian states and Federal District.

Brazilian regions and states	Type of opioid—N (%)^a^				COVID-19 confirmed cases^**^	Number of deaths due to COVID-19^**^	Incidence per 100,000 inhabitants^**^	Mortality per 100,000 inhabitants**	Total opioids per confirmed COVID-19 cases^**^	Total opioids per deaths due to COVID-19^**^
	Fentanyl	Morphine	Remifentanil	Total						
Southeast	1,878,032	87,880	16,985	1,982,897	8,475,071	287,071	9,590	324	0.23	6.90
Espírito santo	24,016	840	40	24,896	600,914	12,796	14,953	318	0.04	1.94
Minas gerais	186,260	11,520	3,815	201,595	2,172,199	55,281	10,261	261	0.09	3.64
Rio de Janeiro	582,956	21,070	NI	604,026	1,308,908	67,697	7,581	392	0.46	8.92
São paulo	1,084,800	54,450	13,130	1,152,380	4,393,050	151,297	9,566	329	0.26	7.61
Northeast	1,358,149	230,970	39,515	1,628,634	4,826,500	117,631	8,457	206	0.34	13.84
Alagoas	189,200	5,020	NI	194,220	239,499	6,268	7,176	187	0.81	30.98
Bahia	279,125	21,420	17,305	317,850	1,241,122	26,992	8,345	181	0.26	11.77
Ceará	312,740	134,500	2,250	449,490	942,351	24,393	10,319	267	0.48	18.42
Maranhão	132,950	8,000	45	140,995	359,227	10,219	5,077	144	0.39	13.79
Paraíba	99,824	27,370	2,000	129,194	444,184	9,380	11,054	233	0.29	13.77
Pernambuco	22,585	7,210	NI	29,795	627,188	19,914	6,562	208	0.05	1.49
Piauí	70,800	10,560	NI	81,360	323,274	7,073	9,876	216	0.25	11.50
Rio grande do norte	160,260	12,200	5,415	177,875	371,278	7,368	10,587	210	0.48	24.14
Sergipe	90,665	4,690	12,500	107,855	278,377	6,024	12,110	262	0.39	17.90
Midwest	458,637	95,740	2,125	556,502	2,318,879	58,012	14,229	356	0.24	9.59
Federal district	81,534	28,770	NI	110,304	512,089	10,745	16,983	356	0.22	10.26
Goiás	100,734	1,070	880	102,684	890,310	23,987	12,685	342	0.12	4.28
Mato Grosso do Sul	168,105	58,990	1,245	228,340	375,571	9,626	13,515	346	0.61	23.72
Mato grosso	108,264	6,910	NI	115,174	540,909	13,654	15,523	392	0.21	8.43
North	794,861	84,550	7,485	886,896	1,857,010	46,729	10,075	253	0.48	18.97
Acre	93,355	32,300	NI	125,655	88,019	1,842	9,980	208	1.43	68.21
Amazonas	67,557	46,410	5,415	119,382	427,304	13,761	10,309	332	0.28	8.67
Amapá	117,410	NI	NI	117,410	123,342	1,989	14,584	235	0.95	59.02
Pará	173,971	NI	280	174,251	595,995	16,713	6,928	194	0.29	10.42
Rondônia	144,089	2,020	1,500	147,609	268,187	6,559	15,090	369	0.55	22.50
Roraima	138,089	1,350	290	139,729	127,010	2,019	20,967	333	1.10	69.20
Tocantins	60,390	2,470	NI	62,860	227,153	3,846	14,442	244	0.28	16.34
South	134,649	60,130	23,890	218,669	4,203,028	94,785	14,021	316	0.05	2.30
Paraná	58,024	14,310	20,560	92,894	1,539,756	40,002	13,466	350	0.06	2.32
Rio grande do Sul	44,885	45,820	Nl	90,705	1,454,824	35,252	12,787	310	0.06	2.57
Santa catarina	31,740	Nl	3,330	35,070	1,208,448	19,531	16,866	350	0.03	1.79

a Data last updated on October 20, 2021; ** Data last updated on October 21, 2021.

NI, not informed.

This data was collected up to October 21, 2021 from the Brazilian Ministry of Health website (Coronavírus Brasil, 2021; Localiza SUS, 2021). NI, not informed.

Interestingly, up to October 20, 2021, Brazil used a total of 5,273,598 opioids in its five regions, with only three different types of opioids available in the SUS, and out of those morphine, fentanyl and remifentanil, accounted for, respectively, 559,270 (10.6%), 4,624,328 (87.6%) and, 90,000 (1.8%) units of opioids used. In our analysis, we also observed that many Brazilian regions with high number of confirmed cases of COVID-19 had few units of opioids available, as the Southeast region, with a 0.23 units of opioids per confirmed COVID-19 case, and the South region, with 0.05 units. Furthermore, taking into account the number of deaths due to COVID-19 and total opioids, these 2 Brazilian regions also presented the lowest index in the country, in which the Southeast had 6.90 opioids units per death due to COVID-19, and the South region accounted for 2.30 (Table 2). These two regions were the most affected by the COVID-19, presenting the highest numbers of cases and deaths, thus their opioid supply should have been increased in order to better manage the COVID-19 cases.

A Brazilian study on hospital analgesic consumption trends carried out from 2011 to 2015 showed that although a noticeable reduction in the public expenditure with analgesia occurred, the costs are still high, so that in the last year analyzed, the total cost of analgesics was 326,515€, and out of this total, 84,545€ were spent with analgesic opioids, which represents approximately 26% of the total cost (Monje et al., 2019).

It seems relevant to observe that Brazil has a lower prevalence of opioid use when compared to the United States of America or the rest of the world. One report from 2004 surveyed more than 15,000 individuals in the first and second grade of high schools and the prevalence of opioid use, at least once in lifetime, was 0.7% (ranging from 0.2% in Rio de Janeiro to 1.4% in Salvador) (Baltieri et al., 2004). Another report interviewed 8,589 Brazilians citizens aged between 12 and 65 years old, and the prevalence of opioid use was only 1.4% (Galduróz and Cebrid, 2003). Finally, the latest report on opioid use in Brazil observed an increased prevalence when compared to previous years, nearly 2.9% of the individuals surveyed stated that they had used opioids at least once in their lives (Krawczyk et al., 2020).

Regarding positive points, the federal government could distribute opioids to all Brazilian states, even with a logistic issue related to great distances and difficult access to some cities in the North. Also, Brazil seems to have a lower prevalence of opioid use disorder. On the negative side, we observed that the federal government distributed a low number of opioids to the Brazilian states, which might have predisposed some regions to shortage of opioids. Also, Brazil did not distribute the opioids taking the COVID-19 cases and deaths into account, which might have had an impact in the outcome of the public health policy of the states.

### 2.4 A Growing Issue: The Dependence of Opioid Worldwide

Although the management of sedation in critical patients in IMV is difficult, it is required during the therapeutical intervention. In high doses or for long periods, its use might result in undesirable effects such as the occurrence of delirium or acute cerebral disfunction, which are considered serious problems for the medical team and the patients’ families. European and American guidelines recommend that, in mechanically ventilated patients, sedation is dosed so that the patient can be awaken easily and at the same time has a competent analgesia, since this might reduce delirium incidence (Analgesia and Sedation in Covid, 2021; EMC, 2021; EMCDDA, 2021; Fehosp, 2021; MS-SUS COVID, 2021; Opioid Basics, 2021; Summary of, 2482, SUS, 2021; Understanding the Epidemic, 2021; WHO Coronavirus, 2021; Page, 2021; Pun et al., 2021). However, chronic and indiscriminate use of opioids might cause dependence as reported in the literature (Kosten and George, 2002). Nevertheless, their use in the COVID-19 pandemic is justifiable for the reasons listed above. Delirium incidence is highly prevalent and prolonged in COVID-19 patients and the use of benzodiazepines along with the absence of the family were modifiable risk factors identified in a multicenter study (Pun et al., 2021).

Patients with opioid dependence might be one of the most affected groups in the pandemic, since they are considered a risk population that is marginalized and require more personalized and constant care (Alexander et al., 2020). Several factors can be associated to the greater impact of the pandemic on this group, for example, a study in the South Africa reported that long periods of lockdown might increase the risk of overdose, since a reduction in the addicted individual’s tolerance occurs. In addition, those individuals might use other substances that are also nervous system depressants such as alcohol and benzodiazepines (Stowe et al., 2020; Thylstrup et al., 2020). Another relevant factor affecting this group is the shortage of methadone and buprenorphine, medicines used to treat opioid use disorder, since the delivery of this medication in the pandemic context might be harmed, which might have led to treatment discontinuation and a return to the use of illegal opioids (Magura and Rosenblum, 2001; Elliott et al., 2017; Sordo et al., 2017; Degenhardt et al., 2019; Gisev et al., 2019).

The United States of America and Europe perhaps are the regions that were most affected by opioid use disorders worldwide, and the COVID-19 might have played an important role in this health issue, as described below.

#### 2.4.1 United States of America

The United States of America faces a growing epidemic of opioid use, in fact, since 2007 statistical data has shown increased death rates related to opioid consumption, with the death of nearly 91 American individuals every day and over 100 million individuals treated in emergency rooms for opioid use (Rudd, 2016; Dayer et al., 2019; Understanding the Epidemic | CDC’s Response to the Opioid Overdose Epidemic | CDC, 2021; CDC WONDER). Also, from 1999 to 2018, the United States of America estimated about 450,000 deaths related to opioid use disorder (Wilson et al., 2020; Seyler et al., 2021). This particular country has a greater variety of opioids than Brazil; therefore, fentanyl and morphine, heroin, oxycodone (OxyContin), methadone, and hydrocodone (Vicodin) are widely used and responsible for the opioid use disorder (Opioid Basics | CDC’s Response to the Opioid Overdose Epidemic | CDC, 2021).

Since 2018, deaths related to drug overdose, including opioid overdose, seem stable, with nearly 70,000 reported deaths per month, however in the early 2020, the number of reported deaths began to rise, reaching nearly 96,000 deaths per month in 2021, in part due to the difficulties the pandemic brought to all American citizens (Vital Statistics Rapid Release, 2021). In the literature, a recent report observed that during the COVID-19 pandemic, fewer drug tests were performed, and unfortunately, the percentage of individuals using opioids (fentanyl, heroin and other opioids) increased significantly when compared to the period prior to the pandemic. For instance, about 4.3% of the individuals tested positive for fentanyl before the pandemic, whereas during the pandemic, this number reached 5.8% of individuals (Niles et al., 2021).

Perhaps, many factors related to the COVID-19 pandemic led to this increased opioid overdose death rate. For instance, there are many barriers related to regulations of essential drugs to treat the opioid use disorder such as methadone and buprenorphine. Also, one way to decrease the SARS-CoV-2 spread was isolation; however, physical and social contact are of utmost importance in the treatment of this disorder (Green et al., 2020). Even before the World Health Organization declared the COVID-19 as a pandemic, several healthcare personnel advocated for the removal of barriers related to the treatment of substance disorder (Samet et al., 2018; Davis and Carr, 2019; Fiscella et al., 2019; Green et al., 2020; Summary of H.R. 2,482 (116th): Mainstreaming Addiction Treatment Act of 2019). Unfortunately, a recent study observed that more than 10% of the methadone clinics in the United States of America and Canada were not accepting new patients due to the COVID-19 pandemic (Joudrey et al., 2021). Several tools can be used to attenuate the impact of the pandemic, as the use of telehealth, the greater flexibility to take the drugs to treat this disorder, and home and online group meetings (Green et al., 2020; National Academies of Sciences, 2020; Mehtani et al., 2021). In fact, telehealth was particularly effective when used as a complement of in-person treatment of selected patients (Cales et al., 2021).

The United States of America faces a growing problem related to drug abuse and the COVID-19 might have hampered the access to opioid use disorder treatment. Also, individuals with opioid use disorder are at increased risk of COVID-19. However, some tolls were implemented in order to attenuate the impact of the pandemic in this particular group, as the use of telehealth to help in the opioid use disorder treatment.

#### 2.4.2 Europe

Although the literature for opioid dependence in Europe is scarce, the findings reported are similar to those found in the United States of America. For example, in 2019, 1.0 million individuals were high-risk opioid users, and 76% of drug fatal overdoses were due to opioids. Also, 26% of the requests for drug treatment were for opioid users (Statistical Bulletin 2021—prevalence of drug use | www.emcdda.europa.eu). Even though it is clear that Europe also faces a growing problem of opioid use disorder, many factors found in the United States of America such as over prescription and use of opioids to manage pain, availability and the cheap cost of opioids, and the lack of accessibility to treatment, are not found in Europe (Volkow et al., 2019; Torrens and Fonseca, 2021). This might have contributed to the fact that dependence levels are not the same in Europe. Although heroin consumption appears to be declining in Europe, maybe due to aging of the population, new synthetic opioids seem to be emerging, as fentanyl and analogues, which constitutes a problem in the COVID-19, since they could be adulterated, falsified, or substituted, thus enhancing their toxic effects (Torrens and Fonseca, 2021).

Few studies evaluated the impact of the COVID-19 in the pattern of drug use in Europe, one Italian study with only 30 subjects observed the levels of heroin use appeared to have decreased during the lockdown period, and right after the end of the lockdown they went back to pre-lockdown levels, this might be explained by the fact that the lockdown provided fewer social interactions in which these individuals were able to use drugs (Gili et al., 2021; EMCDDA Trendspotter briefing: impact of COVID-19 on patterns of drug use and drug-related harms in Europe | www.emcdda.europa.eu). Another study in Finland observed increased use of buprenorphine, amphetamine and 11-nor-9-carboxy-Δ9-tetrahydrocannabinol in 2020, after a short drop in May 2020. Unfortunately, this study did not evaluate opioid use (Mariottini et al., 2021). European individuals with opioid use disorder were more affected by the COVID-19 pandemic, and perhaps, similar measures as those taken in the United States of America could be implemented to attenuate their burden.

Europe also faces a growing opioid addiction problem, and the COVID-19 might have made the access to opioid use disorder treatment more difficult. In that continent, individuals with opioid use disorder are also at increased risk of COVID-19. However, some tools were implemented in order to attenuate the impact of the pandemic in this particular group such as the use of telehealth to help in the opioid use disorder treatment.

### 2.5 Use of Opioids in COVID-19 Patients and Their Adverse Effects

COVID-19 patients with pulmonary impairment also presented other symptoms such as dyspnea, which is a frequent clinical manifestation with repercussions at the physical and psychological levels causing suffering to the patient. Dyspnea mechanisms include: (i increase in the afferent signals of chemoreceptors and mechanoreceptors of the upper airways, lung, chest wall, and muscles of breathing; (ii increase in the respiratory effort sensation, and (iii dissociation between the ventilatory needs and the ventilation capacity (Burki and Lee, 2010)

One of the opioids main mechanisms of action in intubation is the reduction in the metabolic rate and ventilatory needs, decrease in the bulbar reflex to hypercapnia and hypoxia, respiratory center neurotransmission alteration, respiratory sensitization suppression, reduction in the respiratory drive, vasodilation, and anxiety reduction effects (Helms et al., 2020; Kapp et al., 2020; Pun et al., 2021). However, in COVID-19 patients, the strategies to prevent cough and dyspnea with the use of opioids might, many times, postpone the orotracheal intubation procedure and generate severe pulmonary consequences. In addition, the continuous use of opioids was associated with greater risk of patients in intensive care developing delirium, probably due to the fact that higher doses are prescribed, of both sedatives and analgesics, to COVID-19 patients, when compared to patients that did not have this disease (Helms et al., 2020; Kapp et al., 2020; Pun et al., 2021).

A quite trendy term these days is analgosedation, which consists in reaching sedation through the use of opioids before considering sedation through non-analgesic medication (Devlin et al., 2018; Adams et al., 2020). Throughout the pandemic, the use of analgesia and analgosedation was advisable in usual care (Riker et al., 2009; Adams et al., 2020). In the H1N1 virus pandemic, the use of fentanyl was higher in patients with pneumonia caused by the H1N1 virus or with acute respiratory distress syndrome associated with bacterial pneumonia (Olafson et al., 2012), showing that in the context of respiratory virus pandemics such as the current one, opioids are even more demanded. As exemplified, opioids play a relevant role in orotracheal intubation due to several factors. More specifically, fentanyl acts reducing the sympathetic nervous system, mainly reducing arterial pressure and causing respiratory depression (Allen et al., 2021).

However, opioids also present side effects such as diarrhea, hyperalgesia, dysphoria, tolerance and dependence processes, their prolonged use might be associated to immunological system suppression, and high doses of opioids might lead to respiratory depression, exacerbating the poor respiratory condition of those patients (Boom et al., 2012; Franchi et al., 2019; Cismaru et al., 2021). Patients with high doses of opioids might experience hypercapnia and hypoxia, due to the previously mentioned mechanisms, thus contributing to more severe respiratory symptoms (LeGrand et al., 2003; Ataei et al., 2020; Velavan and Meyer, 2020). Chronic use of opioids might lead to the induction of immune cell apoptosis, thymus and splint hypotrophy, and suppression of the proliferation of lymphocytes B and T, in addition to the leukocyte activity (Nabati et al., 2013; Ataei et al., 2020). Unfortunately, the lack of clinical studies on patients infected by the SARS-CoV-2 prevents a thorough evaluation of the possible side effects of the use of opioids during the pandemic (Drożdżal et al., 2020), and an analysis of the impact of the use of these drugs might only be possible after further observational studies are carried out.

Regarding the positive points of opioids in this topic, we could observe that opioids can be used in IMV in order to decrease patients’ pain and the anxiety in respiratory depression. They can also prevent asynchronous breath and reduce the respiratory drive, which is harmful to the patient, and optimize ventilation. However, some negative points were also observed, since the use of opioids might be also associated with increased chest wall rigidity, which can increase the respiratory depression of these patients. Some adverse effects of their use such as diarrhea, hyperalgesia, dysphoria, tolerance and dependence processes were also found, and their prolonged used might be associated with immune system impairment.

## 3 Perspectives

There are several opioids that are important in the COVID-19 management, consequently, the demand for this medication increased exponentially during the pandemic. However, several doubts still remain to be clarified only when further studies are developed, as for example, whether the use of short action opioids can result in greater benefit for COVID-19 patients. Unfortunately, in Brazil, only remifetanil is available and in small amounts, which hampers its implementation, even if it has shown more efficacy in intubation. Additionally, Brazil is going against the pandemic combat, a fact that was observed in different news sources that showed shortage of the ‘intubation kit’ in several hospitals of the country. Even with the efforts of the Health Ministry to buy and distribute this medication and supplements, they were still scarce. On top of that, the investment in drugs without proved efficacy and the dissemination of information related to the ‘COVID kit’, which was proved inefficient against the virus, created costs that could have been better used in the purchase of greater quantities of opioids. It is still uncertain whether the purchase of opioids could or not have had some relevant impact on the number of COVID-19 patients’ deaths. However, if stocks were not so low, those patients could have been assisted with greater comfort.

It is also necessary to evaluate the possible side effects of the use of high doses of opioids in COVID-19 patients. As previously exemplified, opioid continuous use was appointed as an independent risk factor to delirium COVID-19 patients in the ICU. Their indiscriminate use and in high doses in patients in need of mechanical ventilation might result in several side effects that still require further observational studies. For this reason, their use must always be based on the most solid scientific evidence. In addition, high doses of sedation and analgesia in COVID-19 patients are probably related to age and, initially, the affection of a single target organ–lung–which makes sedoanalgesia more difficult. Therefore, it is necessary to manage the combination of several agents (for example, propofol, ketamine, hydromorphone, dexmedetomidine, midazolam, fentanyl, morphine, and remifentanil), increasing the potential risk of side effects such as the increased QT effect, hypertriglyceridemia, hypotension, and delirium, requiring the surveillance of a multi-professional team.

Finally, we must address one of the most important issues is the patients’ addiction to opioid use. Individuals with disorders caused by the use of substances, mainly opioid-related disorders, are at greater risk in the COVID-19 pandemic due to a possible immunological suppression. Opioid users represent a population at high risk of developing critical diseases, either due to complications of underlying conditions that led them to use opioids, or complications caused by the opioids. In addition to overdosing, the use of opioids has been associated to a series of complications that might affect adversely the prognosis of critically ill patients, including myocardial infarction, cerebrovascular accident, and infection. It has become evident that the pandemic had greater impact on marginalized individuals such as drug addicts, mainly those addicted to opioids, since the search for medication and psychological support to treat the addiction was affected by the social isolation measures. Further studies must make a clear distinction whether opioid dependence increased during the pandemic as a result of their more frequent use in hospitals that could lead to addiction, or whether the tools used to fight addiction were affected by the social isolation and restrictive measures, which would lead addicted individuals to a relapse, since both hypotheses are possible.

**FIGURE 3:**
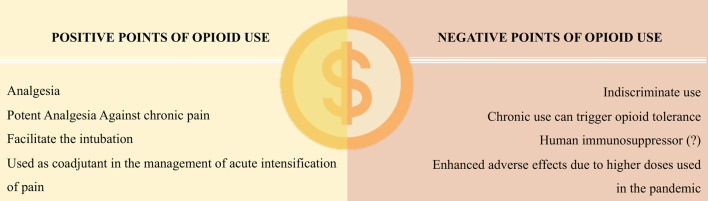
Main risks and benefits associated with the use of opioids.

An informative summary regarding the pros and cons of the opioid use is presented in Figure 3.

## 4 Limitations

The study was carried out based on information made available by the government after a survey on the PubMed-Medline database, which might blur the understanding of the real scenery of opioid use in Brazil, since no hospital was directly evaluated. Governmental data bases as the one used in this study might not be updated or even have lost data, which might hamper the analysis carried out in this study. Despite its importance, the literature for opioids use is still scarce and it is difficult to achieve the highest degree of scientific evidence up to this date regarding all-pros and cons of opioid use during the COVID-19 pandemic. Also, there is discrepancy related to the availability of each drug in different countries, which makes the interpretation of our findings in a broad scenery more difficult.

## 5 Conclusion

In the COVID-19 pandemic scenario, the positive points related to opioids were mainly the occurrence of analgesia, to facilitate the intubation and their use as coadjutant drugs in the management of acute intensification of pain, whereas the negative points included indiscriminate use, the presence of human immunosuppressor response and the enhanced adverse effects due to higher doses of the drug. Also, the importance of rational and individualized use of analgesic hypnotic and sedative anesthetic medication must be considered at all times, mainly in situations of high demand such as the COVID-19 pandemic. Even though necessary, the opioids might be used carefully, since one of their adverse effects is respiratory depression, which can worsen the respiratory symptoms in COVID-19 patients. Finally, the pandemic might have affected not only critically ill patients who needed intubation, but also those with opioid use disorder, who faced a major problem posed by the pandemic isolation measures, thus decreasing their adherence to the drug disorder treatment.
